# Case of Trephining the Spine; Death from Pyæmia; Clinical Remarks

**Published:** 1867-07

**Authors:** 


### CASE OF TREPHINING THE SPINE; DEATH FROM PYÆMIA; CLINICAL REMARKS.

The following is one of very great surgical interest. The question of the advisability of trephining the spine after injuries—an operation recently advocated by Dr. Brown Séquard—was largely discussed at a recent meeting of the Medical and Chirurgical Society, after the reading of a paper by Mr. Berkley Hill. (See *American Journal of Medical Sciences*, April, 1867, p. 538.) For the following report, we are indebted to Mr. Tracey:

J. J., aged 28, while somewhat tipsy, fell off a cart on his right buttock, and was unable to stand or move his lower extremities from that moment. The case was first seen by Mr. Mauder on August 31, three days after the occurrence of the accident, when the patient exhibited more or less loss of sensation below the level of the nipples, and loss of muscular power in the trunk and lower extremities, with constipation and retention of urine. The right buttock was bruised and excoriated, as also was the integument over the angles of the right mid-dorsal region. On examining the spine, the spinous process of the seventh cervical vertebra appeared to be most unusually prominent, and the attempt to move this (though no mobility was appreciable,) gave pain in the region, and also along the back of the patient's right arm. The patient stated that this prominence had existed a long time, and had been caused by carrying bags of sand upon his neck. Respiration was performed chiefly by the diaphragm. The patient was placed upon a water-bed, and beyond attention to his bladder and to the bruise on the buttock, which was gradually converted into a sore, little in the shape of treatment was requisite; but the symptoms gradually changed—incontinence of feces and urine supervened, and the loss of sensation as high as the nipples became complete. He grew weak.

On September 18 he was the subject of a severe cough, accompanied by difficult and copious expiration. He breathed more easily when lying over somewhat on his left side. The urine was highly ammoniacal, and loaded with muco-pus. Dr. Davies was consulted as to the condition of the chest, and advised mist oleosa and brandy. Pulse intermittent.

*19th.*—After consultation with Dr. Ramskill and Mr. Little, Mr. Maunder determined to cut down upon the seat of and explore the injury. Insensibility and loss of volition existed as high as the nipple; the skin over the upper part of the right scapula and along the back of the right arm as far as the elbow, was tender on pressure, and an attempt to move the spine of the seventh cervical vertebra caused pain also. Irritating the feet caused reflex action. Incontinence of feces and of urine as before. The grasp of the right hand was weaker than that of the left, which was strong.

At the operation Mr. Maunder said he was induced to interfere surgically for certain reasons—the physical condition of the spine led him to think that the seventh cervical vertebra, in part or entirely, had been displaced backwards and a little upwards, thus compressing the cord between its body and the laminæ of the first doral. He thought that the cord had not been crushed beyond repair at the time of accident, because there was not from the first a total loss of sensation, but this had gradually become complete by reason of the continued pressure causing loss of temporary function. The operation which he proposed to perform did not, he thought, in itself, entail great risk to life, and was justified by the urgency of the symptoms.

*Operation.*—The patient being on his face, and under the influence of chloroform, (which he bore well,) an incision about three inches in length in the mesian line exposed the spines of the first snd second dorsal vertebræ, and the knife kept close to these readily allowed the muscles to be separated, so as to expose the laminæ also. The muscles were ecchymosed to some extedt. Free bleeding occurred, but no ligature was requisite. The spines of these vertebræ were now cut off at their bases, and the corresponding laminæ were removed by the trephine and bone forceps. The bleeding having ceased, the sheath of the cord was seen at the bottom of the wound, but nothing abormal could be either seen or felt in it. On comparing the interval between the sheath of the cord opposite the laminæ of the third dorsal vertebra, and between it and the laminæ of the last cervical, the space was decidedly greater in the former than in the latter region; but this difference was not sufficient to induce the operator to remove the laminæ of the seventh cervical. The wound was dressed with water-dressing, and the patient returned to bed. Half a grain of extract of belladona was ordered thrice daily.

For some few hours after the operation the patient vomited, and when he had recovered from this, said his legs felt warmer than before the operation. Pain in the arm persisted for two or three days. Cough diminished greatly. On the fourth day subsequent to the operation, he was ordered one-twelfth of a grain of strychnine, thrice daily, and in a day or two reflex action was more readily excited in the lower extremities. On the 29th the cough again became very distressing, and he expired suddenly on October 2.

At a *post mortem* conducted by Dr. Sutton, the medulla spinalis was found more or less pulped opposite the lower border of the seventh cervical vertebra, and this, too, was displaced slightly forward, and its right transverse process was broken. There was not the least trace of inflammatory action in or about the cord and its membranes, but there was ample evidence of pyæmia in the great viscera. The bladder was quite healthy, a condition which Mr. Maunder believed to be due to the attention bestowed upon that viscus by Mr. Salt, one of the dressers. Mr. Maunder suggested that the first attack of cough (before the operation) was also due to the blood-poisoning originating in the bed-sore.—*Med. Times and Gaz.*, Feb. 23, 1867; *Med. News and Library*, Philadelphia.

